# Supplementary material for: Failure of Diphtheria Toxin Model to Induce Parkinson-Like Behavior in Mice
Source: Int J Mol Sci. 2021 Aug 31;22(17):9496. doi: 10.3390/ijms22179496 (PMC8430633; doi:10.3390/ijms22179496)
Supplement: Supplementary file 1 [file ijms-22-09496-s001.zip › ijms-1301637-supplementary.pdf]

Supplementary Tables and Supplementary Figures

Suppl. Table S1 (T-S1)

Genotype	Mice #	Dose DTx	Doses	Dead/Alive
**DTR-flfl**	2	50 ng/g	1	2/0
	1	25 ng/g	1	1/0
	1	12.5 ng/g	1	1/0
	1	6.25 ng/g	1	1/0
	1	3.1 ng/g	1	1/0
	1	1.5 ng/g	1	1/0
	6	100 ng per mouse (≈4 ng/g)	5	4/2
	4	50 ng per mouse (≈2 ng/g)	2	2/2
	9	100 pg/g	5	0/9
**DAT-DTR**	2	50 ng/g	1	2/0
	-	25 ng/g	1	1/0
	1	12.5 ng/g	1	1/0
	1	6.25 ng/g	1	1/0
	1	3.1 ng/g	1	1/0
	1	1.5 ng/g	1	1/0
	8	100 ng per mouse (≈4 ng/g)	5	4/4
	4	50 ng per mouse (≈2 ng/g)	2	3/1
	11	100 pg/g	5	0/11

Suppl. Table S1: DTx dose finding studies. DTx was injected i.p. as a single dose or on consecutive days as indicated. DTx was diluted in 0.9% saline.

Suppl. Table S2 (T-S2)

Sample	Abs. 540 nm	Abs. minus Blank	Endotoxin (EU/ml)	Sample	Abs 540 nm	Abs. minus Blank	Endotoxin (EU/ml)
**Assay water**	0.0606	0		Assay water	0.0608	0	
**0.1 EU/ml**	0.1137	0.0531		0.01 EU/ml	0.097	0.0362	
**0.25 EU/ml**	0.3564	0.2958		0.025 EU/ml	0.1971	0.1363	
**0.5 EU/ml**	0.9243	0.8637		0.05 EU/ml	0.332	0.2712	
**1.0 EU/ml**	1.5971	1.5365		0.1 EU/ml	0.6342	0.5734	
**DTx stock 0.1 mg/ml**	1.7493	1.6887	1.0649	NaCl	0.1114	0.0506	0.0201
**DTx 1.5 ng/g (750 ng/ml)**	0.067	0.0064	0.0529	DTx 1.5 ng/g	0.1798	0.119	0.0183
**DTx 100 pg/g (50 ng/ml)**	0.0675	0.0069	0.0532	DTx 100 pg/g	0.1011	0.0403	0.0319

Suppl. Table S2: Endotoxin ELISA experiments of the stock solution of commercial DTx and at dilutions used for in vivo experiments. The stock solution was 0.1 mg/ml DTx in sterile water, and was further diluted in 0.9% saline 1:100-1:2000 as working solutions used for injections in mice.

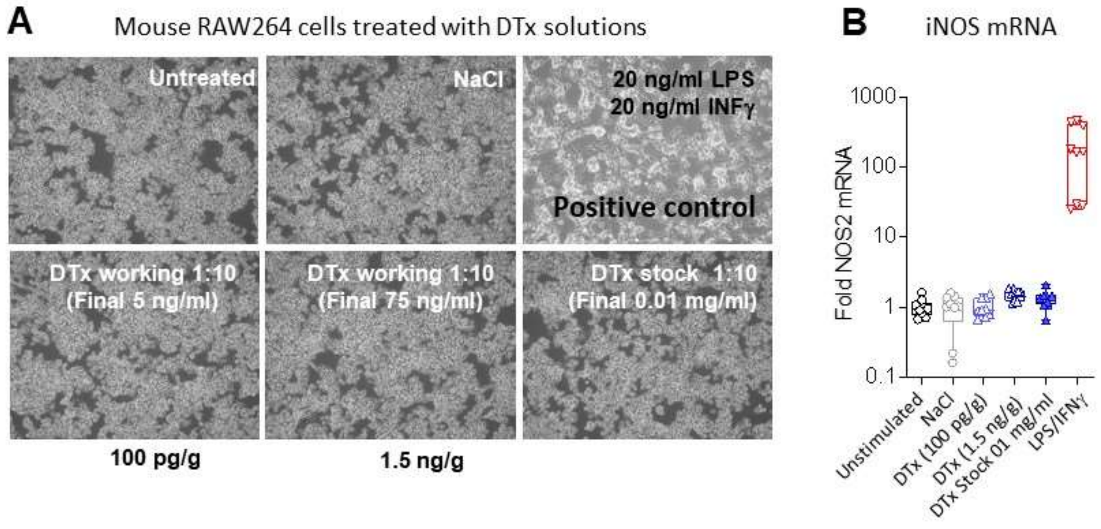

Suppl. Figure S1

Effects of diphtheria toxin (DTx) from Corynebacterium diphtheria in mouse macrophages. DTx is supposed to be non-toxic in rodents and rodents' cells.

A: Morphology of mouse RAW264.7 macrophages in vitro. DTx stock and working solutions had no effect on the viability and morphology of the cells. LPS/IFNγ was used as positive control.

B: Quantitative RT-PCR of inducible nitric oxide synthase (iNOS). iNOS mRNA increased 100fold upon LPS/IFNγ stimulation (positive control) but stimulation with DTX stock or working solutions had no effect.
